# The Condition of the Stomach as a Cause of Health and Disease*Read before I. H. A., June, 1885.

**Published:** 1885-08

**Authors:** C. Pearson

**Affiliations:** Washington


### THE CONDITION OF THE STOMACH AS A CAUSE OF HEALTH AND DISEASE.*

C. Pearson, M. D., Washington.

It is not proposed that this paper shall be an address or lecture, such as a teacher in a college would be expected to deliver to a class of medical students. If there be anything that will make an experienced physician cry out in despair, "Who have sinned, we or our fathers, that we should endure such infliction?" it is to be obliged to sit for half an hour or more listening to a dry, stale paper on some medical topic, perhaps pathology, copied largely from text-books. The more allopathic these, the more profound is the research displayed (!). Yet the lecture from beginning to end, may not contain one practical idea; nothing that years and years ago you did not know to be either true or false.

This custom has become so common of late in the American Institute that it has pretty thoroughly driven out all useful and practical papers, as well as disgusted those members who might have been induced to write them. I may not be able to impart to you any useful information, but will merely state conclusions at which I have arrived from close observation in constant practice for a period of over thirty-five years.

The members of the human family do not live over one-third the length of time they should. The dog comes to maturity in about three years, and five times this or fifteen years is the average length of his life; the horse is at his best at five years and lives twenty-five, and so with all the inferior animals. A man comes to physical perfection at about thirty years of age, and should, therefore, live to be one hundred and fifty. Now what cause can be assigned for his never reaching this age, and only occasionally the one-half of it? Unnatural habits of living, and still more unnatural habits of drugging. The stomach is the great receptacle of health and disease; it may be safely estimated that nine-tenths of all diseases, directly or indirectly, come from gastric derangements.

A very intelligent patient of mine once remarked, "If a man is sick, it is his stomach; if the patient be a woman, it is the uterus." And many greater errors than this have been made even by medical men. He did not take into consideration that uterine derangements, like those of other organs, are often developed from derangements of the stomach. It is true that many of us, like Gloucester, came "into this breathing world

---

*Read before I. H. A., June, 1885.

scarce half made up," and equally true that by inattention to hygienic laws this idiosyncrasy, in very many instances, is increased from a mere predisposition to an organic disease. Hundreds of patients die of what the doctors are pleased to term heart disease, Bright's disease, liver disease, etc., where if any such diseases are present at all, ninety per cent. of them had their origin in gastric ailments. Certain articles of diet, kept up for a length of time, will in some patients produce such marked changes in the urine that the patient, or even the physician, may be led to believe the kidneys are seriously diseased, while at the same time they are in a perfectly healthy condition and doing double duty in separating from the blood impurities that have found ingress through the stomach.

Two or three eggs eaten each day for an equal number of days will often change the urine from a straw to a dark mahogany color. Sugar, potatoes, or milk will often produce a sediment like buttermilk, adhering like lime to the bottom of the vessel; change the diet, leaving these off—for they should never be used where they produce these results—and the urine at once, without the aid of medicine, becomes perfectly normal; continue the diet, and no medicine can do this; as well might you expect to heal a wound with a knife or bullet in it, still keeping up the irritation. Heart symptoms are influenced in the same way. There are probably no agents that produce so many cases of indigestion as tobacco, alcoholic liquors, tea, coffee, and drugs, and consequently so many heart symptoms. Patients will present themselves for treatment for heart disease with their mouths so full of tobacco as to be scarcely able to speak, or the ever-present cigar is only removed long enough to tell their story. Others are never free from a stimulant of some kind, and hence never in a normal condition. Any abnormal action of the heart growing out of the use of these agents, at first perhaps only functional, may in time become organic, and when a fatal termination occurs it will generally be soon after partaking of a hearty meal, or after a fit of indigestion from something recently taken into the stomach. This may not always be from food. Nothing is more indigestible than crude drugs, and they hasten more deaths than they ever did or ever will avert. Alcoholic liquors are but little better. Never in a single instance have I seen any good result from their use, either in health or disease, and how any homœopathic physician can advise or allow his patients to take them while prescribing the high potency of the appropriate remedy, or what he expects to gain from such practice more than the approval of the patient or his friends, is something that to me still remains inexplicable. I tried this stimulating treatment during the first ten or twelve years of my practice, and am fully convinced that my cures were not so prompt with as without them, while debilitating and incurable diseases more rapidly attained a fatal termination. In pulmonary diseases I regard the use of alcohol as suicidal, destroying, as it does, the vitality and life of the patient, robbing the red globules of the blood of the oxygen, which the lungs with their enfeebled powers are laboring to supply. Except in a potentized form, homœopathists should have no use for this agent; its effects are tonical, palliative, and injurious. Throw it where it belongs, with patent medicines and other allopathic deviltries, to the dogs.

Look well, then, to the stomach of your patient. Pay no attention, further than its importance as a symptom, to his craving for certain dishes. Should he follow his inclination in this, his chances for an early funeral will be very fair. I once saw a patient recovering from a fever unable to resist the temptation of drinking a small quantity of sweet cider. Bowel trouble set in, of which he died in a few days. I saw three children convalescing from scarlet fever eat thickened milk, bringing on salivation in less than twelve hours and canker sores through the entire mouth. I once had a patient whom I had treated for vertigo, and who by my direction had left off the use of tobacco and regained his usual health, return to it again, and die from its effects in less than three months. So there are few greater fallacies than that the patient should have what he craves. Many are not aware what disagrees with them until their attention is called to it; they will insist that their stomachs are all right, as they experience no pain in that organ; but they are not aware that owing to the few sensitive nerves the stomach may be fearfully diseased with little or no pain being experienced, except in other organs. A patient of mine who died of cancer of the stomach, found to be such after death, never complained of pain in that region. He had the usual sallow, leather-colored complexion, with the most markedly intermittent pulse I ever saw, so much so that the patient, who was himself a physician, insisted the whole trouble was in his heart.

Cold is thought to be a very common cause of disease, and yet it is questionable whether any one can take cold whose stomach is in a normal condition. Nasal catarrh, either acute or chronic, prevails, perhaps, to a greater extent than any other one disease, and there is nothing more certainly aggravated by irregularities in eating and drinking than this. One hearty meal of highly seasoned food, particularly if pepper be an ingredient, will often start the patient to sneezing in less than two hours, and increase the catarrh in less than twelve. Some patients will tell you they take cold all the time without regard to weather or temperature. Ninety-five per cent. of such cases originate with the stomach.

"Feed a cold and starve a fever" can only be true in the sense that if you feed a cold you will have a fever you will be obliged to starve to get clear of. I do not believe that such a thing as a purely local disease exists; a mechanical injury is not a disease; neither will disease, when not too serious, follow it if the system be kept right with the proper diet and remedies; hence I have no patience with specialists. The healthy action of every organ of the body is dependent to a great extent on the normal state of every other organ, and yet the specialist goes to work as if his favorite organ was entirely independent, doing business strictly on its own account. So he commences at the top, lops off a branch here, and another there, and woe to the abnormal growth or sensitive tooth that may show itself in the way. Of course, these are only results; but no difference, the doctor is expected to do something for his fee, and so he removes them; the root, however, still remains untouched; the cause is kept up through the stomach, and the patient gets clear of his money and is flattered into believing that he is getting better right along—till he dies.

I do not believe that any condiment, any drug, or medicinal substance should ever be taken into the stomach in health or even in disease. The potenized homœopathic remedy is not a drug; it need not and rarely does act through the medium of the stomach, but is taken up by the absorbents of the tongue. It is most remarkable that as the stomach is so generally made a kind of dumping-ground for every indigestible substance, that it should continue to perform its normal functions for so many years. None of the lower animals, with, perhaps, the exception of the ostrich, could endure this one-half the same length of time, though occasionally we find men with stomachs compared with which the ostrich is a dyspeptic. But while it is impossible to swallow poisons and escape their tonical effects, some have more power of endurance or greater powers of resistance, and then from habit the system in health will to some extent adapt itself to abnormal conditions, at first resisting with all its force every invasion by foreign substances, such as tobacco, alcoholic liquors, morphine, etc.; it is finally obliged to make the best terms it can with the enemy, and the patient, for he is then a patient, lives constantly under the influence of a drug from the effects of the reaction of which he immediately feels the necessity of a repetition.

And is it possible that physicians calling themselves homœopathic not only tolerate this abnormal condition in health, but resort to this palliative treatment in disease? There is nothing to justify this, either in science, Homœopathy, or common sense. If your patient should be nervous and wakeful, strike him a stunning blow on the head; this will induce *quiet sleep*. Should a reaction set in and insomnia follow, give him another stroke; that is, keep him under the influence of an opiate, and let the disease look out for itself! As an illustration of this worse than useless treatment, we have only to refer to the cases of Generals Garfield and Grant. It would seem as though their medical attendants were determined to prevent their recovery, if possible. It never seemed to occur to them that the vitality and recuperative powers should be kept up by the only thing that ever did or ever will have this tendency, namely, nourishing diet in a healthy stomach. Without these dietetic and sanitary measures being observed, whatever may be the amount of natural vitality, no one can long survive. Instead, however, of this only source of strength and life, in the case of the two illustrious patients referred to, being scrupulously guarded, it was outrageously and wantonly abused and destroyed by the treatment.

Will any one, by close observation of such a course and its results, ever profit by the lesson? The people may; doctors never do. I am well aware that no system of dietetics can be laid down by which all should be governed. All are not similarly constituted, and the system is often so changed by time that even in the same individual what was once found to agree can no longer be taken with impunity; but if any article of diet, as is sometimes the case owing to some peculiar idiosyncrasy, has always disagreed in health, it is folly to advise or even to allow its use in disease. While man is doubtless an omniverous animal, some can only retain a reasonable share of health by restricting themselves to a very few articles of diet. Persons with prominent canine or cuspidati teeth, as a rule, may be regarded as meat eaters; small, regular incisors will usually indicate a milk diet; but where these teeth are large and prominent fruit should be an important item in the bill of fare; and with large, broad, firmly set molars and bicuspids a coarser diet, such as corn, potatoes, and beans.

Insomnia is becoming the plague of human existence. Am I safe in saying that in the male subject ninety per cent. of all cases originate in the stomach? and apart from uterine and pulmonary derangements, the same proportion in the female patients tells you they must have something to make them sleep. They cannot divest themselves of the allopathic idea that this wakefulness is a cause instead of a result, which all the opiates in the world will never cure; so they become impatient, are not disposed to wait till by the proper remedies and dietetics the cause can be removed, but swallow some narcotic, or, in other words, receive the usual scientific (!) blow on the head, rendering them unconscious, and they congratulate themselves on having found such a skillful doctor, who, if he has luck, will, usually, in from one to three weeks have them out riding—in a hearse! The friends, of course, are satisfied, and only regret that they did not sooner resort to the treatment.

The cure of such cases is a growth; medicine alone will not effect it. You might as well expect a medicine administered to a boy would cause him to grow to be a man in a week. The irritating cause has to be discovered and removed or withheld, and this will usually be found to be something affecting the digestion; then the proper medicine must be used to heal the wound. Only in this way can any disease be permanently cured, whether it be a painful corn or an aching tooth. You may extract the latter and amputate the former, but the cause will still remain and sooner or later manifest itself in some way in other organs.

Some persons say they would rather have shorter lives and indulge in eating and drinking whatever the appetite craves. This, of course, is their privilege. If they wish to commit suicide in this way they can usually be accommodated; nature will permit assistance, but never coercion. Any one who has inherited a large, strong, healthy stomach has had a fortune bequeathed to him of more value than gold, and the profligate who abuses the former is as inexcusable as he who squanders the latter; but how few realize this until it is too late!—how few in after-life can say with trusty Adam,

> "Though I look old, yet I am strong and lusty;
> For in my youth I never did apply
> Hot and rebellious liquors in my blood,
> Nor did not with unbashful forehead woo
> The means of weakness and debility;
> Therefore my age is as a lusty winter,
> Frosty but kindly. Let me go with you;
> I will do the service of a younger man."

